# Chromatin as alarmins in necrotizing enterocolitis

**DOI:** 10.3389/fimmu.2024.1403018

**Published:** 2024-05-31

**Authors:** Colleen P. Nofi, Jose M. Prince, Ping Wang, Monowar Aziz

**Affiliations:** ^1^ Center for Immunology and Inflammation, The Feinstein Institutes for Medical Research, Manhasset, NY, United States; ^2^ Elmezzi Graduate School of Molecular Medicine, Manhasset, NY, United States; ^3^ Department of Surgery, Donald and Barbara Zucker School of Medicine at Hofstra/Northwell, Manhasset, NY, United States; ^4^ Department of Molecular Medicine, Donald and Barbara Zucker School of Medicine at Hofstra/Northwell, Manhasset, NY, United States

**Keywords:** necrotizing enterocolitis, CAMPs, cell-free DNA, HMGB1, eCIRP, TLR4, PRRs, inflammation

## Abstract

Necrotizing enterocolitis (NEC) is a severe gastrointestinal disease primarily affecting premature neonates, marked by poorly understood pro-inflammatory signaling cascades. Recent advancements have shed light on a subset of endogenous molecular patterns, termed chromatin-associated molecular patterns (CAMPs), which belong to the broader category of damage-associated molecular patterns (DAMPs). CAMPs play a crucial role in recognizing pattern recognition receptors and orchestrating inflammatory responses. This review focuses into the realm of CAMPs, highlighting key players such as extracellular cold-inducible RNA-binding protein (eCIRP), high mobility group box 1 (HMGB1), cell-free DNA, neutrophil extracellular traps (NETs), histones, and extracellular RNA. These intrinsic molecules, often perceived as foreign, have the potential to trigger immune signaling pathways, thus contributing to NEC pathogenesis. In this review, we unravel the current understanding of the involvement of CAMPs in both preclinical and clinical NEC scenarios. We also focus on elucidating the downstream signaling pathways activated by these molecular patterns, providing insights into the mechanisms that drive inflammation in NEC. Moreover, we scrutinize the landscape of targeted therapeutic approaches, aiming to mitigate the impact of tissue damage in NEC. This in-depth exploration offers a comprehensive overview of the role of CAMPs in NEC, bridging the gap between preclinical and clinical insights.

## Introduction

Necrotizing enterocolitis (NEC) is a complex inflammatory gastrointestinal disease with devastating sequelae (1). Despite decades of research to identify predisposing factors and effective treatments, NEC remains a leading cause of morbidity and mortality in neonatal intensive care units, especially in low birth weight infants (2). Related mortality rates remain unacceptably high, surpassing 50% in affected infants requiring surgery for NEC (3). Current understanding of the development of NEC in premature infants incorporates factors related to the immature gut, bacterial colonization, and insults causing inflammation and tissue injury (4). Specifically, factors such as increased and exaggerated Toll-like receptor (TLR)-based signaling, compromised microcirculatory perfusion, dysmotility, intestinal barrier breakdown, and impaired enterocyte defenses have been implicated in NEC propagation (5). Unfortunately, momentum in improved pathomechanistic understanding of NEC has suggested few preclinical therapies without revolutionizing the clinical management or outcomes for patients.

An exaggerated inflammatory state underlies the acute phase of NEC pathophysiology (4). Critical immune system components of inflammation are damage-associated molecular patterns (DAMPs) and pattern-associated molecular patterns (PAMPs) binding to pattern recognition receptors (PRRs) and imparting immunologic responses (6). A subset of DAMPs, we named chromatin-associated molecular patterns (CAMPs), have been newly classified as critical endogenous inflammatory promoters (7). CAMPs are molecules derived from the nucleus or related to chromatin that have similar nucleic acid derivations and contribute to tissue injury when released from cells in inflammatory states (7). Molecules that have been classified in this group include extracellular cold-inducible RNA-binding protein (eCIRP), high mobility group box 1 (HMGB1), cell free DNA (cfDNA), mitochondrial DNA (mtDNA), neutrophil extracellular traps (NETs), histones, extracellular RNA (exRNA), messenger RNA (mRNA), and micro RNA (miRNA) (7).

The landscape of research uncovering the role of CAMPs and their receptors in inflammatory diseases is vast (7–10). Evidence demonstrating clinically high levels of many of these molecules in sepsis further supports their translational importance (7). However, despite the elevations of CAMPs in inflammation and the understanding that inflammatory cascades contribute to NEC severity, limited efforts have been undertaken to gain a global understanding of these endogenous factors in contributing to NEC pathogenesis. Thus, this review will discuss the current knowledge of the role of CAMPs in NEC supported by preclinical work and clinical evidence. Furthermore, this review will highlight how therapeutic approaches may be targeted to specific pathways of inflammatory signaling initiated by CAMPs and their downstream pathways.

## CAMPs in NEC and therapeutic implications

### Extracellular CIRP

Cold-inducible RNA-binding protein (CIRP) belongs to a family of cold shock proteins that respond to hypothermic stress (11). Under the physiologic condition, CIRP functions as an RNA stabilizer and chaperone, facilitating translation within cells (11). Upon cellular stress, CIRP may be released extracellularly through active and passive pathways – a process that alters its function (12). Extracellular CIRP (eCIRP) then functions as a CAMP and is capable of binding PRRs and initiating inflammatory sequelae (13). Discovery of the deleterious effects of eCIRP have also revealed elevated levels in adult patients with sepsis and neonates with sepsis (11, 14, 15).

Discovery of the dramatic impact of eCIRP in worsening tissue damage in inflammatory diseases has sparked increasing experimental studies. A preclinical model utilizing intraperitoneal injection of cecal slurry into newborn mouse pups (a model resembling neonatal sepsis with overlap of necrotizing enterocolitis-like injury) demonstrated increasing levels of eCIRP (16). In a murine model of colitis, CIRP-deficient mice had decreased susceptibility to colonic inflammation through decreased expression of proinflammatory cytokines in the colonic lamina propria cells, further implicating CIRP’s role in gastrointestinal inflammatory diseases (17, 18).

eCIRP has also been linked to gastrointestinal diseases in humans. For example, in patients with inflammatory bowel disease (IBD), specifically ulcerative colitis (UC), expression of CIRP in chronically inflamed colonic mucosa was positively correlated with the expression of proinflammatory cytokines, antiapoptotic proteins, and stem cell markers (17, 18). Moreover, CIRP expression was further enhanced in the colonic mucosa of refractory UC (17, 18). A breakdown of humans and preclinical model’s, evidence of all CAMPs in gastrointestinal disease, focusing on NEC, is summarized in **Table 1**.

**Table 1:** Levels of CAMPs in NEC.

CAMPs	Preclinical Models of NEC	Human Neonates with NEC
• **eCIRP**	Mouse model (utilizing intraperitoneal injection of cecal slurry) • Increased systemic levels of eCIRP in septic mice (15)	Human Serum • *Circulating levels elevated in septic neonates* (15)
• **HMGB1**	Mouse models (utilizing formula gavage/hypoxia) • Increased serum HMGB1 in murine NEC (19) • Increased intestinal HMGB1 expression in murine NEC (19–21) Mouse models (utilizing formula and enteric bacteria gavage/hypoxia) • Increased serum HMGB1 in murine NEC (22) Rat models (utilizing formula gavage/hypoxia) • Increased expression of HMGB1 in ileal mucosa (23, 24) Rat models (utilizing formula gavage/LPS/hypoxia) • Increased intestinal expression of HMGB1 (25, 26)	Human Intestines • Increased intestinal HMGB1 expression in human neonates with NEC (19, 24, 27) Human Serum • Increased serum HMGB1 in infants with NEC (22, 28) • Increased serum HMGB1 in Stage II and III NEC compared to Stage I NEC (27) Human Stool • Early fecal HMGB1 levels were predictive of NEC risk (29) Human Genetics • Differences in SNP frequencies in HMGB1 (which may affect HMGB1 expression) were associated with susceptibility and survival in NEC neonates (30)
• **cfDNA**	Mouse models (utilizing formula and LPS gavage/hypoxia) • Increased cfDNA in plasma of NEC mice (31, 32) Pig models (utilizing preterm delivery by cesarean section) • Increased cfDNA in plasma of NEC piglets (33)	Human Intestines • Increased extracellular DNA score in humans with NEC (32) Human Serum • Trends of elevated circulating cfDNA 1-6 days prior to NEC onset (33)
• **NETs**	Mouse models (utilizing formula and LPS gavage/hypoxia) • Markers of neutrophil activation and formation of NETs (by SYTOX orange) increased in NEC (31, 32) Mouse models (utilizing G-CSF/ formula and LPS gavage/hypoxia) • G-CSF increased NEC disease manifestation in mice with increased NETs, whereas ELANE-knockdown (mice incapable of producing neutrophil elastase) were protected from NEC and mortality (34) Mouse model (utilizing intraperitoneal injection of cecal slurry) • Increased circulating platelet-neutrophil interactions (which drive NETosis) in NEC (35) Mouse model (utilizing dithizone/*Klebsiella* (DK) infection) • Increased nucleosomes in NEC ileum from mouse pups (36)	Human Intestines • NETs (visualized with SYTOX orange) were elevated in intestines of neonates with NEC (32) • Neutrophil elastase and markers of NETs increased in neonates with congenital heart disease-associated NEC (37) • NETs visualized within bowel excised from NEC neonates; calprotectin was contained within NETs, whereas NETs-containing calprotectin were not identified in non-NEC (38) Human Serum • Nucleosomes (marker for NET release) were elevated in infants with NEC (36)
• **Histones**	Mouse models (utilizing formula and LPS gavage/hypoxia) • H3cit cells in the intestines were increased in NEC mice (31, 32) Mouse models (utilizing G-CSF/ formula and LPS gavage/hypoxia) • G-CSF increased NEC disease manifestation in mice with increased intestinal H3cit, whereas ELANE-knockdown were protected from NEC, had undetectable H3cit, and had improved survival (34)	Human Intestines: • H3cit was elevated in intestines of neonates with NEC (32) • H4cit3 was elevated in ileum of humans with NEC (36) Human Serum: • Plasma levels of histones (including H4, h3cit) were elevated in human sepsis (39)
• **exRNA and miRNA**	Mouse model (utilizing formula gavage/hypoxia) • Differentially expressed miRNAs in the intestines: miR-141-3p (40); miR-146a-5p (41); miR-200a-30 (42) Mouse model (utilizing formula and bacterial gavage/hypoxia) • Differentially expressed miRNAs in the intestines: miR-301a (43) Mouse model (utilizing formula & LPS gavage/hypoxia) • Differentially expressed in the intestines: LncRNA ENO1-IT1, miR-22-3p (44) Rat model (utilizing formula gavage/hypoxia) • Differentially expressed miRNAs in the intestines: miR-124 (45); miR-222 (46); let-7d-5p (47) • Differentially expressed in the intestines: 53 cirRNA and miRNA interaction networks (48) • Differentially expressed lncRNAs in the intestines: MSTRG.42950, MSTRG.104993, MSTRG.61378, MSTRG.81908 (49) Rat model (utilizing formula gavage/LPS/hypoxia) • Differentially expressed miRNAs in the intestines: miRNAs: miR-27a-5p, miR-187-3p (50); miR-21 (51) Rat model (utilizing rat milk substitute) • Differentially expressed miRNAs in the intestines; miR-34a (52)	Human Intestines • Differentially expressed miRNAs: miR-431 (53); miR-223 (54); miR-301a (43); miR-429/200a/b and miR-141/200c (55); miR-200a-3p and miR-200c-3p (56); miR-223, miR-451, miR-1290, miR-4725-3p, miR-431, miR-4793-3p, miR-21-3p, miR-132, miR-146b-3p, miR-410, miR-375, miR-203, miR-200b-5p, miR-194-3p, miR-200a, miR-215, miR-31, miR-192-3p, miR-141 (57) Human Serum • Differentially expressed miRNAs: miR-1290, miR-1246, and miR-375 (58); miR-34a (52); miR-141-3p (59); miR-21 (51) Human Stool • Differentially expressed miRNAs: miR-223, miR-451a (60) Human Urine: • Differentially expressed miRNAs: miR-376a, miR-518a-3p, and miR-604 (61)

CAMPs, chromatin-associated molecular patterns; NEC, necrotizing enterocolitis; HMGB1, high mobility group box 1; LPS, lipopolysaccharide; SNPs, single nucleotide polymorphisms; cfDNA, cell free DNA; NETs, neutrophil extracellular traps; exRNA, extracellular RNA; miRNA, microRNA; lncRNA, long non-coding RNA; circRNA, circular RNA; H3cit, citrullinated histone 3; H4cit, citrullinated histone 4; eCIRP, extracellular cold-inducible RNA-binding protein.

Advances in understanding the inflammatory footprint of eCIRP have sparked new discoveries of eCIRP-directed therapies. For example, circulating microRNA 130b-3p was found to inhibit eCIRP-mediated inflammation in experimental intraabdominal sepsis, and injection of miR-130b-3p mimic reversed eCIRP-induced inflammation through decreasing its affinity for TLR4 (62). Another therapeutic, C23, an eCIRP-derived peptide, was designed to competitively inhibit binding to the downstream receptor, TLR4 (63). C23 treatment successfully reduced systemic pro-inflammatory cytokines, attenuated markers of tissue injury, and prevented severity of sepsis-induced lung injury in a cecal slurry-based model (63). Another small peptide, M3, was developed to inhibit eCIRP’s interaction with another downstream PRR, triggering receptor expressed on myeloid cells 1 (TREM-1) (16). Treatment with M3 decreased inflammation, reduced lung injury, and improve survival in a murine models of intestinal ischemia-reperfusion and intraperitoneal sepsis (16, 64). Anti-inflammatory therapeutic efficacy of M3 was further replicated in a cecal slurry-based model of neonatal intraabdominal sepsis, whereby M3 improved cardiac dysfunction, attenuated inflammation and improved survival (15).

Milk fat globule-epidermal growth factor VIII (MFG-E8, or lactoferrin) has been implicated as a critical molecule in protecting premature intestines from inflammation and injury through clearance of apoptotic cells (65). Among more recent eCIRP-directed therapeutic advancement has been the creation of a small opsonic peptide, MFG-E8-derived oligopeptide 3 (MOP3) (66). MOP3 was designed to function as an eCIRP scavenger, rather than a competitive inhibitor, thereby “mop-ing” or clearing pro-inflammatory eCIRP from the extracellular compartment (66). MFG-E8 is highly expressed in human breast milk, which carries therapeutic benefits in NEC (possibly through TLR4 inhibition) (67). The recent discovery of MFG-E8’s additional function of eCIRP clearance raises a strong possibility for an additional mechanism whereby MFG-E8 benefits the inflamed intestine through eCIRP-clearance (66). Experimental evidence in murine model of intraperitoneal sepsis supports the mechanism of MOP3-mediated clearance of eCIRP which confers an anti-inflammatory impact, reduction in tissue injury, and a survival benefit (66). A summary of CAMPs and PRR-directed therapies to attenuate gastrointestinal inflammation and NEC is provided in **Table 2**. Future work is needed to elucidate the pathomechanistic impact of eCIRP and potential protein-protein interactions in human NEC.

**Table 2:** Therapeutics Impacting CAMPs and PRR Signaling in NEC.

Targets	Treatment Strategies	Outcome
**eCIRP**	eCIRP-neutralizing antibody	• Attenuated inflammation, reduced organ injury, and improved survival in polymicrobial sepsis [cecal ligation and puncture (CLP)-based] model (11)
	C23 (inhibits eCIRP-TLR4 interaction)	• Reduced systemic pro-inflammatory cytokines, markers of tissue injury, and attenuated sepsis-induced lung injury in murine peritonitis [cecal slurry (CS)-based] model (63)
	M3 (peptide inhibiting TREM-1/eCIRP interaction)	• Decreased tissue injury, systemic inflammation, and intestinal and lung injury, and improved survival in murine models of intestinal ischemia/reperfusion (I/R) injury and intraperitoneal sepsis (CLP-based model) (16, 64) • Improved cardiac dysfunction, attenuated serum, cardiac and pulmonary pro-inflammatory cytokine levels, and improved survival in murine peritonitis (CS-based) model (15)
	MOP3 (MFG-E8-derived oligopeptide 3, scavenges eCIRP)	• Attenuated systemic inflammation, tissue injury, intestinal injury, lung injury, and survival in murine peritonitis (CLP- and CS-based) models (7)
**HMGB1**	Anti-HMGB1 antibody	• Attenuated symptoms of NEC and microvascular features in murine model (formula gavage/hypoxia); rescued NO production through eNOS activation (21)
	Glycyrrhizin (GL, HMGB1 inhibitor)	• Decreased expression of HMGB1 and decreased intestinal injury and inflammation in rat NEC model (formula gavage/LPS/ hypoxia) (25)
	Sodium butyrate (anti-inflammatory, mechanism unknown)	• Decreased intestinal expression of HMGB1, decreased histopathologic injury, and improved survival in murine model (formula gavage/hypoxia/hypothermia) (20)
	Semapimod (macrophage deactivator)	• Inhibited intestinal expression of HMGB1 and reduced intestinal injury in rat NEC model (formula gavage/hypoxia) (24)
	Recombinant human soluble thrombomodulin (rTM, anti-coagulant/anti-inflammatory, mechanism unknown)	• Decreased intestinal expression of HMGB1, reduced NEC injury and inflammation, and improved survival in rat NEC model (formula gavage/LPS/hypoxia) (26)
	Anti-HMGB1 antibody	• Reduced microglial activation and neurologic injury in NEC murine model (formula and enteric bacteria gavage/hypoxia) (28)
**cfDNA, NETs, & histones**	DNAse 1	• Reduced serum cfDNA, decreased markers of neutrophil activation and NETs, and reduced H3cit positive cells; associated with attenuated NEC severity, and improved survival in murine model (formula and LPS gavage/hypoxia) (31) • Decreased pro-inflammatory cytokines, decreased platelet-neutrophil interactions, and improved survival in murine peritonitis (CS-based) model (35)
	Cl-amidine (PAD inhibition)	• Reduced circulating cfDNA, decreased NETs and reduced H3cit positive cells; associated with attenuated intestinal damage and apoptosis, and improved survival in murine NEC model (formula and LPS gavage/hypoxia) (32) • In DK (dithizone/*Klebsiella*)-infection murine model, reduced H4cit, tended to decrease nucleosomes, however, did not significantly alter NEC score and worsened bacteremia and mortality (36) • Decreased pro-inflammatory cytokines, decreased platelet-neutrophil interactions, and improved survival in murine peritonitis (CS-based) model (35)
	nNif (NET inhibitory factor)	• Decreased bacterial killing, decreased pro-inflammatory cytokines, decreased platelet-neutrophil interactions, and improved survival in murine peritonitis (CS-based) model (35)
	STC3141 (small polyanion mCBS, neutralizes extracellular histones and NETs)	• Reduced organ dysfunction and improved survival in rat intraperitoneal sepsis (CLP-based) model (68) • Improved hemodynamics and tissue perfusion, reduced systemic inflammation and tissue injury in sheep fecal peritonitis model (69)
**exRNA and miRNA**	miRNA antagonists and mimics	• miR-222: reversed the decrease of c-kit expression in NEC rat model (46) • miR-34a: decreased intestinal villi damage and inflammatory cytokines in NEC rat model (52) • miR-141-3p: protect intestinal epithelial cells from LPS damage by suppressing RIPK1-mediated inflammation and necroptosis (59) • miR-141-3p: attenuated inflammatory response in NEC mouse model (40) • miR-146a-5p: inhibited NLRP3 inflammasome downstream inflammatory factors in cells (41) • miR-301a: reduced intestinal tissue damage and inflammatory cytokines in murine NEC model (43) • miR-200a-3p: attenuated inflammatory cytokines in intestinal epithelial cells induced by LPS; improved intestinal tissue damage in murine NEC (42) • miR-124: suppressed the intestinal cell apoptosis and histopathologic damage in rat NEC model (45) • miR-148a-3p: reduced NEC incidence and inflammation in murine NEC (70)
	Breast milk-derived exosomes (BM^EXO^) containing miRNAs	• miR-148a-3p, miR-30d-5p, miR-200a-3p in BM^EXO^ decreased intestinal inflammation and improved tight junctions in murine NEC (70)
**TLR4**	C34 (isopropyl 2-acetamido-α-glucoside [C_17_H_27_NO_9_], TLR4 inhibitor)	• Inhibited TLR4 in enterocytes and macrophages, and reduced systemic inflammation in murine endotoxemia and NEC model; inhibited LPS signaling ex-vivo in human ileum from NEC infants (71)
	C34 analog (isopropyl 2-acetamido-α-galactoside, TLR4 inhibitor)	• Suppressed LPS-induced inflammation in IECs and monocytes; greater protection from inflammatory cytokine production in murine endotoxemia than parent structure of C34 (72)
	Human milk oligosaccharides: 2’-fucosyllactose (2’-FL) and 6’-sialyllactose (6’-SL)	• Demonstrated ability to bind pocket TLR4-MD2 complex; reduced TLR4-mediated NF-kB inflammatory signaling in IEC enterocytes; protects against NEC in murine and piglet models; inhibits TLR4 signaling in NEC and in human intestine explants (73)
	Breast milk	• Inhibited activation of TLR4 signaling in enterocytes (via activation of EGFR and GSK3β signaling); inhibited TLR4 signaling and systemic and intestinal inflammation in endotoxemic mice; protected against NEC in murine model (67)
	A18 [aryl hydrocarbon receptor (AHR) ligand]	• AHR ligand, indole-3-carbinole (I3C, of breast milk) administered in pregnancy prevents NEC in mice by limiting TLR4 signaling and expression in intestines; A18 activated AHR and reduced TLR4 signaling in ex vivo human tissue and prevented NEC in mice (74)
	Amniotic fluid	• Amniotic fluid inhibited TLR4 signaling in intestinal epithelium in utero; amniotic fluid-mediated TLR4 inhibition reduced severity of NEC in murine model (via EGFR activation) (75)
	*Lactobacillus rhamnosus* (HN001, probiotic)	• Administration of bacterial DNA inhibited TLR4 signaling in ex vivo human NEC intestines; probiotic attenuated NEC severity in mice and piglet models via activation of TLR9 (76)
**TREM-1**	LP17 (TREM-1 peptide inhibitor)	• Improved survival in endotoxemic mice; improved hemodynamics, attenuated inflammation and improved survival in murine peritoneal sepsis (77)
	LR12 (TREM-1 peptide inhibitor)	• Attenuated colonic inflammation, reduced endoplasmic reticulum stress, and prevented disease-related changes in intestinal microbiota in murine (DSS-induced) colitis (78)
	M3 (peptide inhibiting TREM-1/eCIRP interaction)	• Decreased organ injury and inflammation and improved survival in inflammatory murine models [including intestinal I/R injury, intraperitoneal sepsis (CLP), and neonatal sepsis (CS)] (15, 16, 64)
RAGE	Anti-RAGE antibody	• Improved survival and reduced pathologic small bowel and lung injury in murine intraperitoneal sepsis (CLP-based model) (79)
• cGAS/STING	H151 (small molecule STING inhibitor)	• Attenuated the inflammatory response and reduced tissue injury and mortality in a murine model of intestinal I/R injury (80) • Reduced intestinal injury, intestinal inflammation, gut permeability, and mortality in murine intraabdominal sepsis (CLP-based model) (81)
**NLRP3 Inflammasome**	MCC950 (NLRP3 inhibitor)	• Improved survival, reduced intestinal and neuro-inflammation, and ameliorated intestinal damage in murine NEC model (82)
	YQ128 (selective NLRP3 inflammasome inhibitor)	• Attenuated inflammation in LPS-stimulated monocytes and in vivo murine endotoxemia via selective inhibition of NLRP3 inflammasome (83)
	Bovine milk-derived exosomes	• Exosomes reduced intestinal inflammation in NEC and protected against NF-κB pathway activation and NLRP3 inflammasome activation in murine NEC (84)
	PHLDA1 (pleckstrin homology-like domain family A member 1)	• Inhibited NLRP3 activation (by activating Nrf2) to improve survival, reduce intestinal inflammation and prevent oxidative stress in murine NEC model (85)
	Melatonin (weakens NLRP3 inflammasome activation)	• Improved survival, reduced histopathologic injury, and attenuated intestinal tissue NLRP3 levels in a murine NEC model (86)
	SHMOs (sialylated human milk oligosaccharides)	• Reduced NLRP3 (and TLR4) expression in the ileum of NEC rats; reduced NEC incidence and pathologic damage in rat NEC model (87)
	NS8593 (TRPM7 inhibitor)	• Alleviated TRPM7-mediated NLRP3 inflammasome activation and exhibited protective effects in rat NEC model (88)

CAMPs, chromatin-associated molecular patterns; HMGB1, high mobility group box 1 protein; NEC, necrotizing enterocolitis; NO, nitric oxide; eNOS, endothelial nitric oxide synthase; GL, Glycyrrhizin; LPS, lipopolysaccharide; rTM, recombinant human soluble thrombomodulin; cfDNA, cell free DNA; NETs, neutrophil extracellular traps; H3cit, citrullinated histone 3; H4cit, citrullinated histone 4; CS, cecal slurry; PAD; protein arginine deiminase ; DK, dithizone/Klebsiella; nNif, NET inhibitory factor; ; mCBS, sodium-β-O-Methyl cellobioside sulfate; DSS, dextran sulfate sodium; CLP, cecal ligation and puncture; exRNA, extracellular RNA; miRNA, microRNA; RIPK1, receptor-interacting serine/threonine-protein kinase 1; NLRP3, nucleotide-binding domain, leucine-rich-containing family, pyrin domain-containing 3l; BM^EXO^, breast milk-derived exosomesl eCIRP, extracellular cold-inducible RNA-binding protein; I/R, ischemia/reperfusion ; MOP3, MFG-E8-derived oligopeptide 3; PRRs, pattern recognition receptors; TLR4, toll-like receptor 4; IECs, intestinal epithelial cells; NF-kB, nuclear factor kappa light-chain enhancer of activated B cells; EGFR, epidermal growth factor receptor; GSK3β, glycogen synthase kinase-3β; AHR, aryl hydrocarbon receptor; TREM-1, triggering receptor expressed on myeloid cells 1; RAGE, receptor for advanced glycation end products; cGAS, cyclic GMP-AMP synthase; STING, stimulator of interferon genes; PHLDA1, pleckstrin homology-like domain family A member 1; SHMOs, sialylated human milk oligosaccharides; TRPM7, transient receptor potential melastatin 7.

### High mobility group box 1

High mobility group box 1 (HMGB1) is a nuclear nonhistone, chromatin-binding protein that plays a critical role in DNA replication and repair, regulation of transcription, and nucleosome formation (89). Upon extracellular mobilization, HMGB1 functions as a CAMP, inciting pro-inflammatory responses through binding downstream receptors, resulting in the recruitment of neutrophils, activation of macrophages and endothelial cells, and production of inflammatory cytokines and chemokines (89). HMGB1 is known to be markedly elevated in human sepsis and contributes as a late mediator, leading to greater morbidity and mortality (90). Recent work has focused on uncovering the pro-inflammatory role of HMGB1 in the pathogenesis of NEC.

Murine models utilized to study the condition of NEC have demonstrated increased expression of HMGB1 and increased serum HMGB1 levels (19). Various studies using animal NEC models have replicated the findings of elevated HMGB1, especially in the small intestines (20–26, 28). Remarkably, elevations in HMGB1 expression in murine NEC preceded evidence of intestinal injury (21). Moreover, *in vitro* work has supported that stimulation of intestinal epithelial cells with lipopolysaccharide (LPS, the outer membrane and immunogenic component of gram-negative bacteria) increased expression of HMGB1 (25). Mechanistic work has uncovered that inhibited enterocyte migration by HMGB1 occurs in a TLR4-dependent manner, which was unique to enterocytes, as HMGB1 enhanced the migration of inflammatory cells *in vitro* and *in vivo* (19). In this work, the overall net effect of HMGB1 signaling was a TLR4-dependent increase in cell force adhesion, which accounted for impaired enterocyte migration – demonstrating how TLR4 activation by HMGB1 delayed mucosal repair (19). The overarching impact of HMGB1 in NEC pathogenesis has been related to sequelae of inflammation causing direct intestinal injury; however, additional work has highlighted off-target implications of HMGB1 in systemic manifestations of NEC. For example, a mechanism of gut-lung connection in NEC has been demonstrated whereby intestinal TLR4 activation induced HMGB1 release from intestine, which then activated pulmonary epithelial TLR4 and led to neutrophil recruitment and inflammation in the lung (22). An additional mechanism of gut-brain connection in NEC was suggested, whereby HMGB1 was released from the intestine and activated brain microglia thereby contributing to neurocognitive defects (28).

The translational capacity of preclinical evidence of HMGB1 in exacerbating NEC is rooted in the human condition. Patients with NEC have been shown to have higher expression of HMGB1 than healthy control counterparts (19, 27). Levels of HMGB1 were not substantially different in patients with Stage II or Stage III NEC (when measured in the stool), or patients who were managed medically or surgically (when measured in the serum) (91, 92). Nevertheless, HMGB1 levels in stool samples were higher in preterm neonates compared to full-term neonates with birth weight less than 2.5 kg, and early HMGB1 fecal levels were predictive of NEC risk, thus implicating stool HMGB1 as a potential clinical biomarker in this disease (29). Further evidence of the clinical relevance of HMGB1 is provided by differences in single nucleotide polymorphisms (SNP) frequencies in HMGB1 (which may affect expression) that were associated with susceptibility and survival prognosis in neonates with NEC (30).

Targeting of HMGB1 has been explored to attenuate NEC pathophysiology. *In vitro* testing of enteral miconazole was effectively able to reduce HMGB1 in Caco-2 cells under hypoxic stress (93). In a rat NEC model, inhibition of HMGB1 expression improved intestinal inflammation in NEC (25). Another study used administration of semapimod (a macrophage de-activator), which inhibited HMGB1 upregulation and partially protected against intestinal injury in experimental NEC (24). Separately, the molecule recombinant human soluble thrombomodulin (rhTM) binds and antagonizes HMGB1, thus addressing elevated tissue levels of HMGB1 in preclinical NEC, and provided an anti-inflammatory impact (26). Treatment of NEC pups with an anti-HMGB1 neutralizing antibody attenuated intestinal microvascular features and symptoms of NEC; however, this was not found in eNOS^-/-^ mice (endothelial nitric oxide synthase), suggesting HMGB1 inhibition increased intestinal perfusion in an eNOS-dependent manner as a mechanism for attenuating NEC (21). Sodium butyrate has also been shown to decrease intestinal HMGB1 expression and translated to improved intestinal inflammation and survival outcomes in murine NEC (20). This work suggests that the amplification of cascades involving HMGB1 may be inhibited by butyrate treatment through the TLR4/NF-κB pathway, and through this mechanism inhibited intestinal inflammation in NEC (20). Evidence revealing the important role of HMGB1 in NEC supports further investigation into HMGB1-directed therapies to prevent and attenuate NEC pathogenesis.

### Cell free DNA

During active and passive forms of cell death, cellular DNA exits the cell and becomes immunogenic extracellular or cell-free DNA (cfDNA) (94). cfDNA including pathogen-derived CpG and damage cell-released nuclear or mitochondrial DNA subsequently activate the immune system through association with DNA sensors on the cell surface and sensors within immune cells (94). Importantly, cfDNA has been reported to contribute to the severity and length of the inflammatory response, but also represents a biomarker for inflammatory diseases (94). For example, plasma levels of cfDNA are elevated and linked to increased mortality in humans with sepsis (95). Increasing work has implicated the role of cfDNA in exacerbating NEC pathophysiology.

Evidence supporting the translation of cfDNA inflammatory pathways in NEC is rooted in preclinical modes. First, a preclinical model replicating NEC utilizing preterm pigs revealed increased plasma levels of cfDNA and neutrophil-associated proteins, and the abundance of these levels correlated (33). In a murine NEC model, cfDNA was significantly elevated in the serum of pups upon NEC induction, which was associated with increased intestinal injury and inflammation in the intestines (31). Serum cfDNA was also shown to correlate with NEC macroscopic and microscopic manifestations in mice, and increases appeared to occur in a time-dependent manner (32). A related form of extracellular DNA, mitochondrial DNA (mtDNA) has also been studied in murine NEC, where mtDNA release from the intestine into circulation was increased in experimental NEC conditions (96). Findings supporting the role of increased aberrant cfDNA in NEC have been translated to human infants. Notably, cfDNA and DNase have been suggested as potential biomarkers for diagnosing early and late-onset neonatal sepsis in preterm infants (97). Specific to NEC, plasma cfDNA tended to be elevated in pre-term infants 1 to 6 days before NEC diagnosis compared to controls (33). Furthermore, elevated levels of cfDNA and related markers were detected in humans with NEC, and demonstrated to be similar to those in mice subjected to NEC insult, highlighting the relevance of cfDNA and the translational potential between species (32).

Finally, the importance of understanding the role of cfDNA is further emphasized in evidence supporting it as a valuable therapeutic target. For example, treatment with inactivated DNase 1 (a cfDNA directed therapeutic probe) not only reduced the rate of NEC induction in mice but improved NEC-related survival in the model (31). Furthermore, treatment with Cl-amidine (causing protein arginine deiminase (PAD) inhibition) reduced tissue damage, inflammation, and mortality in murine NEC (32). Given that DNase and PAD inhibition also reduce NETosis, there is likely overlap given that extracellular strings of DNA comprise NETs content. As such, the interplay of these mechanisms and therapeutic strategies will be discussed further in the respective NETs section.

### Neutrophil extracellular traps

Extracellular traps (ETs) are comprised of extracellular meshes of DNA structures, granule proteins, and chromatin, which may be aberrantly released upon cellular activation (98). ETs released by neutrophils are among the most widely studied and referred to as neutrophil extracellular traps (NETs), which may aid in pathogen clearance (9). Once NETs are released extracellularly, various proteins can adhere, including CAMPs and other components of primary and secondary granules and can confer bactericidal activity (99). However, the immune footprint impartment by NETs can be two-fold. Although NETs serve a role in protecting hosts from infectious diseases, pathologic NET release can exacerbate inflammation and increase tissue injury (99). For example, although NET formation may be a protective response in early sepsis, excessive NET formation may induce thrombosis and propagate multisystem organ failure in severe sepsis (9). Given NETs are composed of cfDNA, much of the work uncovering the role of cfDNA in NEC overlaps with the study of NETosis. Despite the challenges associated with detection of NETs (due to their fragility and turnover), related work has shed new light on the role of NETosis in the propagation of NEC.

NETosis has been shown to be elevated in mice upon NEC induction compared to controls (32, 36). In these cases, SYTOX orange allowed for direct visualization of NET formation. The importance of neutrophils and NETs was further supported by a novel animal model of NEC whereby mice stimulated to increase neutrophil concentrations had exacerbated NEC pathology (34). The role of NETosis in preterm infants with NEC has also been explored (100–102). Deficiency in NET formation identified in preterm infants has been linked to a reduction in extracellular bacterial killing *in vitro*, which may impact the susceptibility of neonates in particular to infectious processes, including NEC (101). In human tissues collected from neonates with NEC, visualization of NETosis (by SYTOX orange) was increased in a similar manner to that seen in murine NEC (32). Other work has demonstrated that NEC neonates had increased fecal calprotectin compared to NEC rule-out neonates, and calprotectin within bowel excised from NEC neonates was contained within NETs (38). On the other hand, NETs-containing calprotectin were not identified in non-NEC surgically obtained bowel, suggesting calprotectin may be released as a result of neutrophil infiltration and NET formation within the intestines in the pathogenesis of NEC (38).

Clinically, subtypes of NEC have been differentiated in infants with congenital heart disease, where it is proposed that the pathophysiology is more directly impacted from ischemia/reperfusion injury sustained from inadequate blood supply to the superior mesenteric artery, resulting in poor bowel perfusion (37). In these cardiac-related NEC cases, neonatal intestinal tissue had increased staining for neutrophil elastase and citrullinated histone 3 (H3), as well as increased systemic neutrophils, compared to inflammatory NEC patients. This points to the possibility that NETs partially mediate the component of ischemia/reperfusion-induced injury in NEC (37). Despite evidence suggesting the involvement of NETs in the pathogenesis of NEC, overlap of NET influence in other gastrointestinal inflammatory diseases complicates its use as a biomarker due to limited specificity (103). Nevertheless, further research is required to elucidate the mechanistic components of NETosis and its impact on human development of NEC.

The dual role of NETs in the gut complicates studies targeting NETosis to impart therapeutic benefit (104). Positively, NETs released in the gut may reduce the translocation of bacteria and support the healing of the intestinal mucosa (104). On the other hand, excessive NET formation can negatively impact intestinal barrier function by directly damaging the intestinal mucosa (105, 106) The majority of studies targeting NETosis in experimental NEC (using LPS, formula, and hypoxia-based models) demonstrate a positive impact with strategies inhibiting excessive NET release. For example, treatment with inhibitors of NETosis including DNase 1 and Cl-amidine, nNIF (NET-inhibitory factor), and models using ELANE-knockdown (animals without neutrophil elastase) were protective against NEC severity, demonstrating improved inflammatory profiles and prolonged survival (31, 32, 34, 35). On the other hand, conflicting evidence has been raised with the use of Cl-amidine to target NETs in an alternative model. Specifically, in a dithizone/Klebsiella model of NEC, treatment with Cl-amidine did not impact circulating neutrophils or affect intestinal injury scores; rather, Cl-amidine resulted in increased systemic inflammation, bacterial load, organ injury and mortality (36). This work suggested the possibility that NETs may be involved in the innate immune defense by preventing systemic bacteremia, and thus may have some protective role (36). Altogether, these studies further highlight the nuanced physiologic trade-off of NETosis. Furthermore, contradictory data across models suggests that the overall impact of NETosis may not only be time-dependent but also is likely disease- and model-specific and largely dependent on the level of enteric bacterial translocation. Increased attention to the balance of NET formation and its relation to disease progression will be vital to delineate effective therapeutic strategies that prevent and treat intestinal insults in NEC.

### Histones

Histones are cationic, intra-nuclear proteins that serve to maintain the normal structure of chromatin (107). In states of cellular stress and in a process similar to DNA, histones and DNA-bound histones (nucleosomes) are released into the extracellular space (as in the case of necrosis, apoptosis, and ETosis) (108). Extracellular histones, which may exist as free histones or DNA-bound, are then capable of propagating inflammation, although different forms may induce different cytotoxicity and proinflammatory signaling (108). Previously, intravenously injected histones were found to be lethal in mice, and plasma levels of histones were increased in human sepsis (39, 109). Further, it has been demonstrated that histones may cause cellular injury through a TLR4-dependent manner – a receptor that has been highlighted as critical component to initiate NEC (110). Thus, the role of extracellular histones in inflammatory diseases, including NEC, warrants exploration.

Given that limitations of available techniques complicate differentiation between free versus DNA-bound extracellular histones, much of the work elucidating the role of histones in NEC stems from studies of NETosis, where histones may be released among components of ETs (108). For example, citrullinated histone H3 (H3cit), considered a marker of NETs, was elevated in mice upon NEC induction (32). H3cit scores in NEC intestines were also elevated in experimental models (31). Findings in animal models have also been translated to infants. In peripheral blood of septic infants, citH3-DNA levels were increased and with strong specificity, suggesting a possible early biomarker for neonatal sepsis (111). Specifically in NEC, levels of H3cit were elevated in human NEC samples (32). Furthermore, levels of nucleosomes were elevated in the serum of infants with NEC stage II and above compared to gestational age-matched controls, further suggesting the role of extracellular histones in NEC pathogenesis (36).

Translational evidence supporting the targeting of histones stems from studies of NETosis in NEC. For example, DNase 1 attenuated NEC severity and reduced H3cit scores in mouse intestines in experimental NEC (31). PAD4 functions to citrullinate histones in activated neutrophils, enhancing chromatin unraveling and NET formation (112). Using PAD4 inhibitor to treat experimental NEC effectively reduced H3cit levels, along with reducing inflammation, intestinal injury, and mortality in NEC (32). Newer small polyanions such as STC3141 (sodium-β-O-Methyl cellobioside sulfate, mCBS) have been developed specifically to neutralize extracellular histones and NETs (68, 69). Promising early preclinical evidence utilizing STC3141 demonstrated effective neutralization of histones and NETs while reducing organ dysfunction and improving survival in animal models of intraperitoneal sepsis (68, 69). Altogether, this evidence supports the role that extracellular histones may play in exacerbating NEC pathogenesis and the potential to target aberrant histones as a strategy for reducing inflammation in the treatment of NEC.

### Non-coding RNA

Endogenous RNA can engage nucleic-acid sensing PRRs and initiate inflammatory sequelae (113). It is well recognized that nucleic-acid and RNA sensing pathways are relevant beyond microbial sensing, and have been documented to contribute to various inflammatory disease models (113). Non-coding RNAs have been previously implicated in gastrointestinal disease pathology, including IBD and colitis (48). miRNAs have been implicated to impact biological functions in gastrointestinal diseases, such as through affecting cell apoptosis, cell proliferation, intestinal epithelial barrier function, and inflammation infiltration by post-transcriptional gene silencing (114). As elevated circulating levels of miRNAs have also been demonstrated in septic patients previously, increasing attention has been paid to studying these molecules in neonatal gastrointestinal inflammatory disease, including NEC (115).

There is a growing body of literature uncovering the impact of RNAs in NEC, which are summarized in **Table 3** (116). Intestinal epithelial cell death and breach of mucosal integrity is an important initiating process in NEC development. Certain RNAs have been implicated in this process, for example, circular RNAs (circRNA) were differentially expressed in an animal NEC model and impacted apoptotic signaling and death receptor activity, thereby contributing to cell death (48). Many differentially expressed miRNAs have been identified in NEC impacting intestinal epithelial cell death, including, miR-200a-3p, miR-141-3p, miR-431, and miR-21 (40, 42, 51, 53, 59). In searching for early NEC biomarkers using differential microarray analysis of NEC infants plasma, three miRNAs (miR-1290, miR-1246, and miR-375) emerged as being highly sensitive (0.83) and specific (0.92) in NEC detection (58). In one of the largest studies, miR12-90 was also noted to be more specific to NEC, as this was found to be distinguishable from non-NEC, sepsis cases (58).

**Table 3:** RNAs identified in the pathogenesis of NEC.

miRNAs in NEC	Signaling pathways	Impact
**↑ in NEC:** miR-431 (53)	**Downregulates:** ESRRG, LGR5, NFKB2, PLA2G2A, PRKCZ **Downregulates:** FOXA1	**↑** intestinal injury and inflammation
	**Downregulates:** HNF4A, PRKCZ	**↑** tight junction dysregulation
**↑ in NEC:** miR-1290 miR-1246 miR-375 (58)	**Downregulates:** FOXA1	**↑** intestinal injury and inflammation
**↑ in NEC:** miR-124 (45)	Downregulate: ROCK1, MYPT1	**↑** intestinal injury, apoptosis, and inflammation
**↑ in NEC:** miR-181a-5p miR-124-3p miR-194-5p miR-362-3p (49)	**Upregulates:** TLR4, TORC2, Notch, P53, mTOR	**↑** intestinal injury and inflammation
**↑ in NEC:** miR-27a-5p (50)	Effects PRKCA, PLCB3, VANGL1, SFRP1	Deranged intestinal renewal
**↑ in NEC:** miR-222 (46)	**Downregulates:** c-kit	**↑** intestinal injury and inflammation
**↑ in NEC:** miR-223 (54, 60)	**Downregulates:** NFIA	**↑** intestinal injury and inflammation
**↑ in NEC:** miR-34a (52)	**Downregulates:** SIRT1	**↑** intestinal injury and inflammation
**↑ in NEC:** miR-146a-5p (41)	**Upregulates:** NLRP3	**↑** intestinal injury and inflammation
**↑ in NEC:** miR-301a (43)	*Unknown*	**↑** intestinal injury and inflammation
**↑ in NEC:** lncRNA MSTRG.42950 lncRNA MSTRG.104993 (49)	Interaction with miRNAs miR181a-5p, miR-124-3p, miR-194- 5p, and miR-362-3p TLR4 TORC2 Notch P53 mTOR	NEC development
**↑ in NEC:** miR-451, miR-4793-3p, miR-21-3p, miR-431, miR-1290 (57)	TLR4/NF-kB AP-1/FOSL1 FOXA1 HIF1A	**↑** intestinal inflammation and hypoxia/oxidative stress
**↓ in NEC:** lncRNA MSTRG.61378 lncRNA MSTRG.81908 (49)	Interaction with miRNAs miR181a-5p, miR-124-3p, miR-194- 5p, and miR-362-3p TLR4 TORC2 Notch P53 mTOR	NEC development
**↓ in NEC:** miR-203, miR-31, miR-194-3p (57)	TLR4/NF-kB AP-1/FOSL1 FOXA1 HIF1A	**↑** intestinal inflammation and hypoxia/oxidative stress
**↓ in NEC:** miR-141-3p (40, 59)	**Upregulates:** RIPK1; MNX1	**↑** intestinal injury and inflammation
**↓ in NEC:** miR-187-3p (50)	Effects PRKCA, PRKCB, PPAR	Deranged intestinal renewal
**↓ in NEC:** miR-429/200a/b miR-141/200c (55)	**Upregulates:** VEGF1, FLT1, KDR, SELE, HGF	**↑** alterations in intestinal microcirculation and perfusion
**↓ in NEC:** miR-200a-3p miR-200c-3p (56)	**Upregulates:** KDR, BDNF, YWHAG, YWHAE, YWHAB	**↑** alterations in intestinal microcirculation and perfusion
**↓ in NEC:** let-7d-5p (47)	**Upregulates:** LGALS3 (TLR4/NF-kB)	**↑** intestinal injury and inflammation
**↓ in NEC:** miR-200a-3p (42)	**Upregulates:** RIPK1	**↑** intestinal injury and inflammation
**↓ in NEC:** miR-21 (51)	**Downregulates:** PTEN/GSK-3β **Upregulates:** PI3K/AKT	**↑** cellular apoptosis and intestinal necrosis
**↓ in NEC:** miR-22-3p (44)	*F. Nucleatum/* LncRNA ENO1-IT1/miR-22-3p/IRF5	**↑** inflammation
**↓ in NEC:** miR-148a-3p (70)	**Upregulates:** p53 **Downregulates:** SIRT1	**↑** intestinal injury and inflammation

miRNA, microRNA; NEC, necrotizing enterocolitis; RIPK1; receptor-interacting protein kinase 1; MNX1, motor neuron and pancreas homeobox 1; ESRRG, estrogen related receptor gamma; LGR5, leucine rich repeat containing G protein-coupled receptor 5; NFKB2, nuclear factor kappa B subunit 2; PLA2G2A, phospholipase A2 group IIA; PRKCZ, protein kinase C zeta; FOXA1, forkhead box A1; HNF4A, hepatocyte nuclear factor 4 alpha; ROCK1, rho associated coiled-coil containing protein kinase 1; MYPT1, myosine phosphate targeting family; RIPK1: receptor interacting serine/threonine kinase 1; TLR4, toll like receptor 4; TORC2, CREB regulated transcription coactivator 2; P53, protein 53; mTOR, mechanistic target of rapamycin kinase; PRKCA, protein kinase C alpha; PRKCB, protein kinase C beta; PPAR, peroxisome proliferator activated receptor; PLCB3: phospholipase C beta 3; VANGL1, VANGL planar cell polarity protein 1; SFRP1, secreted frizzled related protein 1; VEGF, vascular endothelial growth factor; FLT1, fms related receptor tyrosine kinase 1; KDR, kinase insert domain receptor; SELE, selectin E; HGF: hepatocyte growth factor; BDNF, brain derived neurotrophic factor; YWHAG, tyrosine 3-monooxygenase/tryptophan 5-monooxygenase activation protein gamma; YWHAE, tyrosine 3-monooxygenase/tryptophan 5-monooxygenase activation protein epsilon, YWHAB: tyrosine 3-monooxygenase/tryptophan 5-monooxygenase activation protein beta; LGALS3, galectin 3; NF-kB, nuclear factor kappa B; c-kit, receptor tyrosine kinase; NFIA, nuclear factor I A; SIRT1, sirtuin 1; NLRP3, NLR family pyrin domain containing 3; PTEN, phosphatase and tensin homolog; GSK-3β, glycogen synthase kinase-3 beta; PI3K/AKT, phosphatidylinositol 3-kinase/protein kinase B; lncRNA, long non-coding RNA; IRF5, interferon regulatory factor 5; AP-1, activator protein 1; FOSL1, FOS like 1; HIF1A, hypoxia inducible factor 1 alpha subunit; NOD2, nucleotide binding oligomerization domain containing 2.

Along with intestinal epithelial cell death in the early pathogenesis of NEC is the hallmark of exaggerated inflammation, with the importance of PRRs (like TLR4) being emphasized. miRNA’s linked to inflammatory sequelae in NEC include miR-124, miR-223, miR-222, miR-34a, and miR-146a-5p (41, 45, 46, 52, 54). In looking at the interaction of long non-coding RNAs (lncRNA), differentially expressed lncRNAs were identified in a rat NEC model (upregulated MSTRG.42950 and MSTRG.104993, and downregulated MSTRG.61378 and MSTRG.81980) which bound target miRNAs (miR181a-5p, miR-124-3p, miR-194- 5p, and miR-362-3p) to modulate TLR4 signaling in NEC inflammation (49). Let-7d-5p, a member of a miRNA family involved in self renewal, was downregulated (with higher expression of LGALS3) in a rat NEC model, and implicates the anti-inflammatory role of let-7d-5p through TLR4-axis in NEC (47).

As intestinal epithelial cells are subjected to injury and inflammatory insult, a key component in NEC pathology is the impaired regenerative ability of the gut subjected to repetitive stress. Differentially expressed miRNAs have been identified in NEC linked to this process, including miR-27a-5p, rno-miR-187-3p, miR181a-5p, miR-124-3p, miR-194- 5p, and miR-362-3p (49, 50). Another critical component of NEC pathogenesis is the role of impaired gut perfusion. Differentially expressed miRNAs in NEC have been implicated in altering blood flow, including miR-429/200a/b and miR-141/200c, miR-200c-3p and miR-22a-3p, and a network of dysregulated miRNAs (miR-31, miR-451, miR-203, and miR-4793-3p) (55–57). Some miRNAs have been linked to other components of NEC, including dysbiosis. For example, the bacteria Fusobacterium nucleatum was found to be abundant in patients and animal models of NEC, and that miR-22-3p was a target of this bacteria (through LncRNA ENO1-IT1), suggesting this axis as a target in NEC (44).

Critically important in miRNA discovery is understanding the translational capacity to target differentially expressed miRNAs. Many of these human-based studies have been corroborated in animal models (43). Furthermore, miRNA mimics and antagonists have demonstrated ability to impact downstream signaling and NEC outcomes by targeting respective miRNAs (40, 42, 43, 45, 46, 52, 70). Some limitations for clinical translation have been identified. For example, increased expression of miRNAs (miR-223 and miR-451a) were identified in human NEC stool samples, however considerable overlap in levels between NEC and non-NEC patients may interfere with diagnostic and therapeutic capabilities of targeting miRNAs (60).

Increasing attention has been paid to small carriers of nucleic acids, including miRNAs, lncRNAs, and circRNAs, contained in extracellular vesicles (EVs) (117). In one study, EVs in the urine of premature neonates with NEC had differentially decreased expression of miRNAs (miR-376a, miR-518a-3p, and miR-604), and transduction molecules associated with these miRNAs (including TP53 and RPS15) were transcriptionally reduced in an animal model, suggesting crossover between models and human disease (61). Stem-cell derived exosomes, which contain RNAs, have also been explored as a therapeutic option to ameliorate NEC (118). Moreover, EVs containing RNAs in milk have been implicated to play a beneficial impact in NEC (119); however, greater study is needed to determine the nature of these vesicles cargo and their exact role in the mechanisms underlying NEC development.

## Targetable signaling pathways

### Toll-like receptors

Various CAMPs have been identified as ligands of toll-like receptors (TLRs). Specifically, evidence supports that HMGB1, eCIRP, and extracellular histones are ligands of TLR4; cfDNA, mtDNA, extracellular histones, and HMGB1 are ligands of TLR9; extracellular histones and HMGB1 are ligands of TLR2; and exRNAs are ligands of TLR’s 3, 7, and 8 (10). TLRs function to sense PAMPs and DAMPs (including CAMPs) through their N-terminal extracellular leucine-rich repeats (120). When immune cells are exposed to the ligands of TLRs, they exhibit intracellular signaling cascades that can induce the expression of a variety of overlapping and unique genes involved in immune and inflammatory responses (121). Dramatic progress has been made in the last decade in improving our understanding of TLRs, which has largely been driven by a desire to understand the pathogenesis of clinical inflammatory conditions – that mainly being septic shock (122). As TLR4 has been widely recognized as the main receptor of LPS, it has garnered great attention in studying pathophysiology of infections and pro-inflammatory diseases.

The connection of TLRs to NEC pathophysiology has largely hinged around TLR4 (123). The importance of TLR4 in NEC pathobiology roots in the discovery that the expression levels of TLR4 are higher in premature intestines compared to full-term intestines, in both human infants and other model species (124, 125). Activation of TLR4 by extracellular ligands has been shown to contribute to NEC development (125, 126). The focus on the role of TLR4 has been paid to the initial recognition and host response to gram negative bacteria (127) the Specifically, the mechanism of TLR4 involvement in NEC is described whereby the intestines of premature infants become colonized with gram-negative bacteria, which activate TLR4 and trigger pro-inflammatory downstream signaling cascades (128). This leads to increased enterocyte apoptosis and necroptosis, impaired mucosal healing, and enhanced pro-inflammatory cytokine release (129, 130). This injury contributes to a ‘leaky gut barrier’ where increased bacterial translocation can further activate TLR4 on the mesenteric endothelium leading to vasoconstriction by endothelial nitrate (71, 131). Subsequent reduction in blood flow and reduced intestinal perfusion contributes to worsened intestinal ischemia and necrosis (131). TLR4 signaling also impacts T cell profiles, as increased recruitment of pro-inflammatory Th17 lymphocytes at the expense of anti-inflammatory regulatory T cells worsens the exaggerated inflammatory response in the bowel (132).

TLR4 signaling has been linked to NEC pathogenesis through additional mechanisms. For example, the role of dysbiosis has been a long-recognized component of NEC pathology (133). Much work has suggested that the functional expression of TLRs is critical in the dynamic interaction between the host epithelium and the microbiota that enables successful intestinal adaptation to the commensal microbiota (134–136). Specified bacterial changes (such as increases Proteobacteria and decreases in Firmicutes and Bacteroidetes), have been further implicated in NEC development and are rich in TLR4 ligands (137). Remarkably, the focus on TLR4 activation has remained on activation by bacterial ligands; however, notable ligands of TLR4 include endogenous activators, including CAMPs. Despite the link of CAMPs in activating TLR4 and contributing to other inflammatory diseases, there is a gap in understanding how these various molecules converge into existing proposed pathways. Likely, activation of TLR4 in NEC development occurs through the binding of both bacteria and endogenous CAMPs in concert.

A vast body of work supports the interplay of TLR4 in NEC. In an experimental NEC model, mice lacking TLR4, specifically on the intestinal epithelium, were protected from NEC development and associated inflammatory sequelae (138). Further animal work supports the mechanism by which TLR4 activation disrupts the enteric nervous system of the newborn intestine, and subsequent enteric glial loss triggers dysmotility and initiation of early NEC pathogenesis (139). The discovery of activating mutations in TLR4 signaling pathways seen in infants with NEC further supports the translation of these findings to the human condition (140, 141).

In discovering the interplay of TLR4 activation and NEC development, harnessing mechanistic discoveries for therapeutic utility is critical. Pharmacologic inhibitors of TLR4 have been developed and tested to attenuate NEC in experimental conditions. A family of TLR4 inhibitors has been identified to reduce intestinal inflammation in experimental NEC (72). Specifically, C17H27NO9 (C34), a 2-acetamidopyranoside is a readily absorbed and nontoxic oligosaccharide that inhibits TLR4. C34 demonstrated great promise by preventing NEC incidence in mice and piglets and decreased TLR4 signaling and inflammation ex vivo in resected ileum from infants with NEC (142). This body of work suggests similar analogs of TLR4 inhibitors may hold therapeutic value and improve clinical treatments for NEC.

Other mechanisms of TLR4 inhibition to improve outcomes in NEC have been identified. For example, breast milk has long been recognized to be protective against NEC development. A potential link is provided in that breast milk was found to be a potent inhibitor of TLR4 signaling by preventing glycogen synthase kinase 3β activity (67). It is possible that MFG-E8 (lactoferrin) provided in breast milk inhibits the ability of LPS binding protein to adhere to TLR4, and thereby inhibits LPS-stimulated TLR4 signaling (143). The human milk oligosaccharide 2’-fucosyllactose and 6’-sialyllactose was also found to protect against NEC by inhibiting TLR4 (73). Downregulation of TLR4 signaling by breast milk may reverse the inhibition of intestinal stem cell proliferation and mucosal healing, which are themselves inhibited by TLR4 (67, 138). Activation of aryl hydrocarbon receptor (AhR) either by its ligand indole-3-carbinol or by breast milk components also prevented experimental NEC through inhibition of TLR4 signaling (74).

The use of probiotics to prevent NEC development has also been explored. CpG-containing bacterial DNA (which uniquely bypassed the potential adverse effects of live bacterial treatment), was found to be effective against experimental NEC in mice and piglets through inhibition of TLR4 (and activation of TLR9) (76). Despite numerous trials demonstrating a reduction in the incidence of NEC and prevention of mortality with probiotic administration in preterm infants, there has subsequently been conflicting evidence and concern raised regarding the safety and efficacy of routine probiotic use, preventing ubiquitous uptake in the U.S (144, 145). Although controversy remains over the use of probiotic supplementation in infants at risk for NEC, probiotics may have significant impacts on not only TLR-signaling, but other CAMP-driven pathways that may contribute to NEC development. Further studies are needed in this regard, with a focus on probiotic impact on CAMPs and the associated mechanisms that influence gastrointestinal inflammatory phenotypes in NEC. Another strategy in NEC treatment utilized simulated amniotic fluid and yielded promising results, likely through targeting mucosal protection conferred by amniotic fluid, which is rich in growth factors and exerts anti-TLR4 effects (75). Currently, the clinical trial NCT02405637 is evaluating the efficacy of synthetic amniotic fluid in preventing NEC among very low birth weight infants (146). Together, these targeted strategies provide mechanistic understanding and potential preventative strategies against NEC linked through TLR4.

The convergence of a multitude of factors identified in NEC pathogenesis around TLR4 has accelerated focus on this bacterial receptor; however, other toll-like receptors can recognize CAMPs and impact immune balance and responses. For example, TLR9 is found on endosomes and recognizes nucleic acids derived from pathogens and self- damaged cells (122). Originating from bacterial DNA, TLR9 is a cell receptor for unmethylated CpG dinucleotides. TLR9 acts as an antagonist of TLR4 and has been shown to be protective against NEC severity (147). Specifically, the TLR9/TLR4 link has been demonstrated whereby murine and human NEC intestines had decreased TLR9 and concurrently increased TLR4 expression (148). Targeting TLR4 (through enteral administration of adenovirus expressing mutant TLR4) resulted in increased expression of TLR9 in intestines and reduced NEC severity in neonatal mice (148). Furthermore, TLR9 activation with CpG-DNA reduced NEC severity, and genetic knockdown of TLR9 exacerbated NEC severity in mice, further supporting the role of TLR9 in NEC pathogenesis (148).

Recently, the dysregulation of TLR repertoire in NEC has been further elucidated. In a murine NEC model and consistent with previous studies, TLR4 was shown to be increased up to 1.7 fold in NEC intestinal tissue, (and slight increases in TLR8 and TLR13, although not significant) (4). Expression of other TLRs was decreased in jejunal and ileal tissue from NEC pup models, including TLR1 (up to 58% decrease), TLR3 (42% decrease), TLR5 (74% decrease), TLR6 (93% decrease), TLR9 (70% decrease), TLR11 (94% decrease), and TLR12 (92% decrease) (4). There were no detectable differences of TLR2 and TLR7 expression between NEC and dam-fed pups (4). Together, these data suggest the modulation of intestinal baseline TLR repertoire, including their interrelation, as mechanisms underlying NEC susceptibility and development. Future research is needed to untangle the multifactorial components of TLR activation, including by CAMPs in addition to bacteria, and interaction of receptors and ligands in NEC pathophysiology.

### Triggering receptor expressed on myeloid cells-1

Although all ligands have not been completely identified, the CAMPs, HMGB1 and eCIRP, have been identified as ligands of triggering receptor expressed on myeloid cells-1 (TREM-1) receptor, along with LPS, a stimuli of TLR4 (10, 149). TREM-1 is a PRR which can be upregulated and amplify immune responses in inflammatory states (150). Exaggerated inflammation mediated through TREM-1 activation is considered a critical contributor to the pathophysiology of sepsis through promoting release of inflammatory cytokines and chemokines (151). Elevated expression of TREM-1 has been identified on immune cells of septic patients, along with circulating levels of cleaved soluble extracellular TREM-1 (sTREM-1) (152, 153). Increasing discovery of TREM-1 involvement in acute inflammation has linked TREM-1 upregulation and activation to numerous diseases, including those affecting the gastrointestinal system.

TREM-1 signaling has been studied in pathologic states of bowel inflammation. In experimental mouse models of colitis and patients with IBD, intestinal TREM-1 expression was upregulated and correlated with disease severity (154). sTREM-1 was also positively correlated with Crohn’s disease activity index and clinical activity indexes of UC in IBD patients compared to healthy controls (155). Although the vast majority of resident intestinal macrophages lack expression of TREM-1 in physiologic conditions, aberrant immune reactions against luminal antigens and alarmins may be disease-promoting factors, contributing to TREM-1 upregulation and immune imbalance in gut inflammatory disorders.

TREM-1 has been investigated as a potential therapeutic target in neonatal inflammatory conditions (156). For example, LP17 was one of the first peptides developed to inhibit TREM-1, and demonstrated the ability to improve survival in endotoxemic mice (77). LP17 has been suggested as a possible therapeutic target in neonatal disease, including sepsis (156). Blocking TREM-1 with this antagonist peptide (LP17) also attenuated clinical severity and histopathologic damage in experimental model of murine colitis (154). Pharmacologic inhibition of TREM-1 with different therapeutic peptide, LR12, along with genetic knockdown of TREM-1, was also successful in protecting mice from severity of colitis (78). A newer inhibitory peptide, M3, was designed specifically to interfere with TREM-1 activation by eCIRP (64). M3 treatment effectively attenuated disease severity in cecal slurry-induced sepsis and improved cardiac function and survival in the same model (15, 64). Although the intraperitoneal sepsis induced by cecal slurry captures some elements of NEC pathophysiology, further research is needed to uncover the role of TREM-1 in exacerbating NEC development and the potential of TREM-1 directed therapies to prevent excessive inflammatory damage and disease severity.

### Receptor for advanced glycation end-products

Microbial products, as well as endogenous CAMPs (cfDNA, exRNA, and HMGB1) have been shown to be ligands of the receptor for advanced glycation end-products (RAGE) (10). Due to its ability to recognize a broad range of structurally diverse ligands, including endogenous and exogenous molecules, RAGE has been classified as a PRR and is a key regulator of the innate immune response (157). The upregulation of RAGE in gut-specific inflammation follows previous systemic findings in sepsis and accompanies upregulation of its ligands (158, 159). For example, RAGE expression was found to be increased in the inflamed small bowel of Crohn’s disease patients compared to non-inflamed bowel (160). A possible mechanism may involve recruitment of neutrophils by HMGB1 activating RAGE (161). Further, a specific RAGE polymorphism was suggested to protect from structuring Crohn’s disease, possibly by increasing the levels of soluble RAGE (sRAGE) which neutralizes pro-inflammatory mediators (162).

The translational capability of targeting RAGE to reduce gut inflammation has been investigated. For example, in *in vitro* studies, RAGE blockade in stimulated immune cells isolated from Crohn’s disease mucosa decreased secretion of inflammatory cytokines (160). In a more translational model, administration of sRAGE (which may act as a decoy receptor) was effective at suppressing inflammation and gut injury in a murine colitis model (163). Moreover, an anti-RAGE antibody demonstrated ability to prolong survival and reduce pathologic organ injury in a murine model of intraperitoneal sepsis (79). To our knowledge, only one study has linked RAGE to NEC pathology (24). Specifically in investigating HMGB1, expression of RAGE was found to be upregulated (along with its respective ligand) in the ileal mucosa of NEC rats compared to breast-fed controls (24). In this work, treatment with a macrophage deactivator (semapimod) in rat NEC model reversed NEC-induced upregulation of RAGE (along with HMGB1), possibly through cytokine inhibition and blockade of MAP kinase (24). Although direct targets of RAGE have not been studied in NEC specifically, collective work revealing RAGE upregulation in inflamed intestines support its possible use as a therapeutic target.

### cGAS-STING

Certain CAMPs, including cfDNA, mtDNA, and NETs, have demonstrated capacity to activate cyclic GMP-AMP (cGAS) and the cyclic GMP-AMP receptor, stimulator of interferon genes (STING) (164–166). Although cGAS can detect pathogenic DNA to trigger an innate immune response, cGAS can also be activated through endogenous DNA, including CAMPs, resulting in the production of secondary messenger, cGAMP. cGAMP then activates the endoplasmic reticulum-localized adaptor protein, STING, which when activated induces a strong type 1 interferon response (167). This pathway has emerged as a key mediator of inflammation in states of infection, cell stress, or tissue damage (167). Overactivation of cGAS-STING has emerged as a key pathway promoting inflammation in gastrointestinal diseases (168).

Animal models have been utilized to probe the influence of cGAS-STING signaling in gut inflammation. For example, in a murine model of enterocolitis (manifested by loss IL-10), experimental enterocolitis was less severe with cGAS-deficiency and was completely abrogated with STING-deficiency (169). In a model of DSS-induced colitis, STING protein expression was increased, and STING agonist worsened colitis whereas STING-knockdown reduced the severity of colitis (170). Furthermore, constitutive activation of STING in mice led to intestinal dysbiosis and spontaneous colitis, of which the altered microbiome was found to exacerbate intestinal inflammation through STING ubiquitination and activation (171). Recent evidence has highlighted the importance of this pathway in human diseases, as patients with UC were found to have elevated colonic levels of STING (171).

Insights into the molecular biology and impact of the cGAS-STING pathway have allowed for the development of selective small-molecule inhibitors targeting the cGAS–STING axis to treat diseases, including reducing gut inflammation (167). For example, H151 was developed as a small molecule inhibitor of STING, and has demonstrated ability to reduce tissue injury and mortality in a murine model of intestinal ischemia/reperfusion injury (80). The beneficial impact of H151 on the gastrointestinal tract was recapitulated in a murine model of intraabdominal sepsis by reducing intestinal injury, intestinal inflammation, gut permeability, and preventing mortality (81). Although no previous studies have investigated the role of cGAS-STING signaling in NEC, future work is needed to identify the impact of this pathway, either through activation by microbial pathogens or endogenous CAMPs, and the potential for targeted therapies to similarly attenuate this gastrointestinal inflammation.

### Intracellular sensors: RIG-I, AIM2, and NLRP3

CAMPs are also capable of activating cytosolic sensors. For example, exRNA and cfDNA can activate retinoic acid-inducible gene I (RIG-I) (172). RIG-I detects microbial DNA and endogenous RNAs and DNAs and induces a pro-inflammatory type 1 interferon response (172). cfDNA has been shown to impart signaling through recognition by cytosolic sensor absent in melanoma 2 (AIM2) (10). AIM2 is a member of innate immune sensors that can detect aberrant self-DNA or pathogenic foreign DNA and lead to inflammasome assembly and cytokine secretion (173). Histones, exRNA, and eCIRP can activate a similar cytosolic sensor, nod-like receptor family pyrin domain containing 3 (NLRP3) (10). Nucleotide-binding oligomerization (NOD)-like receptors (NLRs) constitute a class of PRRs contained within inflammasomes, and include both AIM2 and NLRP3 (174). Triggering of these intracellular signaling pathways (and their convergence) leads to the induction of numerous cytokines and chemokines and has been studied in relation to exacerbating inflammatory diseases (175).

Preclinical work has highlighted the relevance of these cytosolic sensors in gut inflammation (176). For example, generation of RIG-I-deficient mice demonstrated a colitis-like phenotype and increased susceptibility to DSS-induced colitis (177). Furthermore, RIG-I-knockdown caused colonic inflammatory infiltrate, decreased size and number of Peyer’s patches, and dysregulated T cell activation (177). AIM2 has also been linked to gut inflammation. For example, AIM2 deficient mice were found to develop severe dysbiosis-mediated colitis through loss of commensal regulation, highlighting the interaction between cytosolic sensors, microbial colonization, and immune balance (178). Furthermore, in patients with IBD, AIM2 was expressed in macrophages and epithelial cells of the small and large intestines, suggesting a role for AIM2 in gut-related inflammatory diseases (179, 180).

Although RIG-I and AIM2 have not been studied in NEC specifically, the NLRP3 and inflammasome pathway has been considered in this disease. In an animal model of NEC, NLRP3 mRNA levels were significantly raised in the intestinal tissues of rats (181). This was further corroborated in that murine NEC intestines had upregulated NLRP3 (82). NLRP3-deficient mice also exhibited decreased intestinal injury and improved mortality (182). Importantly, the NLRP3 inflammasome enzymatic protein caspase-1 and its downstream inflammatory factors are not only increased in NEC intestinal samples from mice, but are also increased in NEC samples from humans (41). Intestinal lamina propria of NEC patients had high NLRP3 (182). Increases in NLRP3 expression were found in patients with NEC in other studies as well (87, 88).

The translational implication of these activated pathways has been revealed within studies of NLRP3. For example, a second-generation NLRP3 inhibitor, YQ128, selectively inhibited NLRP3 and attenuated inflammation in a murine model of endotoxemia (83). Inhibition of NLRP3 with therapies like MCC950 and PHLDA1 (pleckstrin homology-like domain family A member 1) also improved intestinal inflammation and survival in experimental NEC (82, 85). Blocking calcium efflux mediated by the membrane protein, transient receptor potential melastatin 7 (TRPM7) also reduced damage in experimental NEC by inhibiting NLRP3 inflammasome activation (88). The miRNA mimic (miR-146a-5p) inhibited NLRP3 inflammasome downstream inflammatory factors in cells and is another possible relevant factor in inflammatory signaling in NEC (41). Moreover, treatment with bovine milk-derived exosomes in an animal model of NEC attenuated lung injury by reducing NLRP3 inflammasome activation and NF-κB signaling (84).

The crosstalk between PRR-pathways is implicated in work evaluating multiple signaling mediators. For example, in enterocytes, suppressor of cytokine signaling 3 (SOCS3) protected against NEC through by modulating NLRP3/AIM2 inflammasome activation in a TLR4-dependent manner (183). Melatonin also demonstrated improved survival in murine NEC model and attenuated mRNA and protein levels of both NLRP3 and TLR4 (86). Additional work revealed that an improved inflammatory profile in NEC by inhibiting HMGB1 was through inhibiting NLRP3 via TLR4 and NF-κB signaling pathways (25). Sialylated human milk oligosaccharides (SHMOs) supplementation ameliorated the elevation of TLR4 and NLRP3 in the ileum of NEC rats (87). Finally, it was suggested that Cronobacter sasakazakii infection contributes to increased inflammasome (NLRP3) and TLR4-mediated intestinal damage in NEC models (184). A summary of these various therapeutics that impact signaling transduction pathways in NEC is further included in **Table 2**. Many of these studies lack robust mechanistic understanding yet identify mediators of convergent pathways and implicate an interplay of many pro-inflammatory signaling cascades.

**Figure 1:**
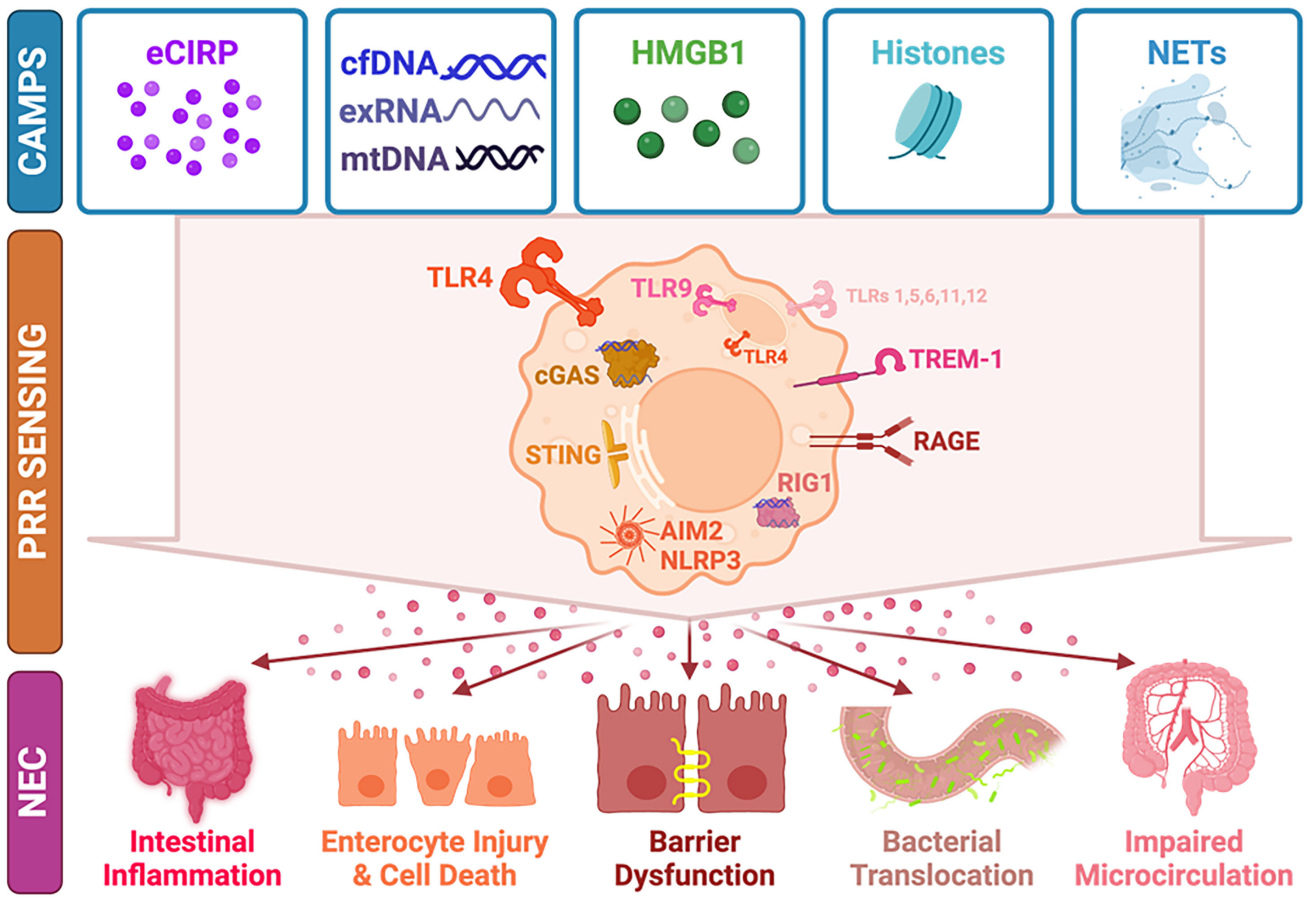
CAMPs, PRRs, and the Development of NEC. CAMPs, including eCIRP, cfDNA, exRNA, HMGB1, histones, and NETs are released upon cellular stress. CAMPs are recognized and activate downstream PRR pathways including TLRs (especially TLR4 and TLR9), cGAS-STING, RAGE, RIG-1, AIM2, and NLRP3. Activation of immune sensors leads to release of pro-inflammatory mediators and causes intestinal inflammation, enterocyte injury and cell death, intestinal barrier dysfunction, bacterial translocation, and impaired microcirculation, all contributing to NEC development. CAMP, chromatin-associated molecular patter; PRR, pattern recognition receptor; NEC, necrotizing enterocolitis; eCIRP, extracellular cold-inducible RNA binding protein; cfDNA, cell-free DNA; exRNA, extracellular RNA; mtDNA, mitochondrial DNA; HMGB1, high mobility group box 1; NETs, neutrophil extacellular traps; TLR, toll-like receptor; TREM-1, triggering receptor expressed on myeloid cells-1; RAGE, receptor for advanced glycation end-products; cGAS, cyclic GMP-AMP (cGAS), STING, stimulator of interferon genes; RIG-1, retinoic acid-inducible gene-I; AIM2, absent in melanoma-2; NLRP3, nod-like receptor family pyrin domain containing-3.

Although there are still gaps in understanding the roles of cytosolic sensors such as RIG-1-like receptors (RLRs) and NLRs in NEC development specifically, cellular perturbations during inflammation in NEC results in the release of host CAMPs (or microbes) which may then be sensed and activate downstream pathways. An overview of CAMPs and their contribution to NEC pathobiology though PRR sensing is provided in **Figure 1**. Further research is needed to better understand these signaling events in NEC and their crosstalk to facilitate targeted anti-inflammatory therapeutic development.

## Conclusions and future directions

Immune imbalance and exaggerated inflammatory signaling exacerbate NEC development. Thus, an improved understanding of the inciting inflammatory factors and signaling pathways is vital to uncovering NEC pathophysiology and developing effective treatments. First, improved identification of the critical ligands in early intestinal inflammation is needed. As it is understood that NEC onset occurs only once the premature gut has been colonized by bacteria, it is likely that microbial pathogens are early players involved in sensing by PRRs to initiate inflammation (128). However, additional endogenous factors, such as CAMPs, have been implicated to play a key role in causing inflammation to run amok. Likely, these endogenous factors released upon initial tissue injury then contribute to tipping the immune balance towards uncontrolled inflammatory insult in the gut, subsequent systemic inflammation, and devastating end-organ injury. Although ongoing research highlights the importance of CAMPs and their downstream signaling cascades in this disease, further work is needed to investigate therapeutically targeting these pathways to improve outcomes. Moreover, translational work uncovering the interplay of dysbiosis and host CAMPs on PRR sensing will guide differentiated strategies for prevention and treatment. Will maintenance of a homeostatic, anti-inflammatory microbiome more effectively help prevent NEC development, while targeting CAMPs and signaling pathways provide a better anti-inflammatory treatment strategy once inflammatory aberrancy begins? Understanding the time course and interplay of the multifactorial components of NEC pathophysiology will drive strategic breakthroughs to revolutionize needed clinical treatments for this devastating disease.

## Author contributions

CN: Conceptualization, Data curation, Formal Analysis, Investigation, Methodology, Software, Validation, Visualization, Writing – original draft, Writing – review & editing. JP: Investigation, Resources, Supervision, Visualization, Writing – review & editing. PW: Conceptualization, Funding acquisition, Project administration, Resources, Supervision, Validation, Visualization, Writing – review & editing. MA: Conceptualization, Formal Analysis, Funding acquisition, Investigation, Methodology, Project administration, Resources, Software, Supervision, Validation, Visualization, Writing – review & editing.
